# Brain natriuretic peptide levels have diagnostic and prognostic capability for cardio-renal syndrome type 4 in intensive care unit patients

**DOI:** 10.1186/cc7878

**Published:** 2009-05-15

**Authors:** Sunghoon Park, Goo-Yeong Cho, Sung Gyun Kim, Yong Il Hwang, Hye-Ryun Kang, Seung Hun Jang, Dong-Gyu Kim, Young Rim Song, Young-A Bae, Ki-Suck Jung

**Affiliations:** 1 Division of Pulmonary, Allergy and Critical Care Medicine, Department of Internal Medicine, Hallym University Sacred Heart Hospital, 896 Anyang, Kyunggi-do, 431-070, Republic of Korea; 2 Division of Cardiology, Department of Internal Medicine, Hallym University Sacred Heart Hospital, 896 Anyang, Kyunggi-do, 431-070, Republic of Korea; 3 Division of Nephrology, Department of Internal Medicine, Hallym University Sacred Heart Hospital, 896 Anyang, Kyunggi-do, 431-070, Republic of Korea; 4 Department of Radiology, Hallym University Sacred Heart Hospital, 896 Anyang, Kyunggi-do, 431-070, Republic of Korea

## Abstract

**Introduction:**

Limited data are available regarding the diagnostic and prognostic utility of brain natriuretic peptide (BNP) in patients with chronic kidney disease (CKD) in the intensive care unit (ICU) setting.

**Methods:**

All patients with CKD and a serum creatinine (Cr) of 2.0 mg/dl or higher admitted to the ICU between January 2006 and September 2007 were enrolled in this study. The CKD group was divided according to the presence or absence of acute decompensated heart failure (ADHF) into CKD + ADHF and CKD - ADHF groups, respectively. Other patients with ADHF having low Cr (<1.2 mg/dl) in the coronary care unit were also recruited as a control group during the same period. BNP levels at the time of admission (admission BNP) were compared amongst these groups. We then sought to determine whether BNP levels could predict the outcome in patients with CKD.

**Results:**

Of 136 patients with CKD for whom data were available, including 58 on dialysis (42.6%), 81 (59.6%) had ADHF and their estimated glomerular filtration rate (eGFR) was 12.8 ± 7.3 ml/min/1.73 m^2^. BNP levels at admission were 2708.6 ± 1246.9, 567.9 ± 491.7 and 1418.9 ± 1126.5 pg/ml in the CKD + ADHF, CKD - ADHF and control groups (n = 33), respectively (*P *= 0.000). The optimal cutoff level in patients with CKD was 1020.5 pg/ml (area under the curve = 0.944) to detect ADHF from the receiver operating characteristic (ROC) curve. This level was not associated with in-hospital mortality, all-cause death or a composite event (all-cause death and/or new cardiac event). However, a borderline significant association was observed with new cardiac events (hazard ratio (HR) = 4.551; *P *= 0.078) during the follow-up period (521.1 ± 44.7 days). Furthermore, continuous variables of BNP and BNP quartiles were significantly associated with new cardiac events in the multivariate Cox model (HR = 1.001, *P *= 0.041; HR = 2.212, *P *= 0.018).

**Conclusions:**

The findings suggest that the level of BNP at the time of admission may be a useful marker for detecting ADHF and predicting cardiac events in patients with CKD in the ICU setting.

## Introduction

Cardiovascular disease is a leading cause of death in patients with chronic kidney disease (CKD), for whom the cardiovascular mortality rate is 15 to 30 times higher than in the general population [1,2]. Acute decompensated heart failure (ADHF) occurs in approximately 40% of patients with CKD and is a strong independent risk factor for mortality [2,3]. Recently, the importance of heart-kidney interaction has received widespread attention, and a new classification of the cardio-renal syndrome (CRS) with five subtypes has been proposed. In this classification, CRS type 4 is characterised by a condition of primary CKD contributing to decreased cardiac function, ventricular hypertrophy and/or increased risk of adverse cardiac events [4,5].

Brain natriuretic peptide (BNP), a bioactive cardiac neurohormone secreted from the ventricular myocardium in response to myocardial stretching and volume overload, has diagnostic and prognostic utility in patients with ADHF who do not have CKD [6-9]. BNP levels are also frequently elevated in patients with CKD who have volume overload or concomitant left ventricular dysfunction (i.e., CRS type 4), but the precise mechanisms of these effects are poorly understood [10-12].

In a stable outpatient setting, several studies have shown that the BNP level may be a good predictor for cardiac events and mortality [13,14]. However, no previous studies have focused on the significance of BNP in patients with CKD admitted to the intensive care unit (ICU), although such patients in the ICU are expected to have high BNP levels and high cardiovascular event and mortality rates.

Therefore, the present study was performed to investigate whether BNP level at admission may be useful in detecting ADHF and predicting cardiovascular outcome in patients with CKD admitted to the ICU.

## Materials and methods

### Study population

After obtaining approval of the protocol from the institutional review board, anonymous data from electronic medical records for all adult patients (≥ 18 years old) admitted to the ICU during the 21-month period from January 2006 to September 2007 were reviewed. Informed consent was waived due to the retrospective nature of the study.

For the CKD groups, patients with high serum creatinine (Cr) levels (≥ 2.0 mg/dl) and with a documented medical history of CKD were included in the study. This CKD group was divided into CKD + ADHF and CKD - ADHF groups according to the presence or absence of ADHF, respectively. Patients who were admitted after cardiopulmonary resuscitation or surgery and those who remained in the ICU for less than 24 hours until death or were discharged against medical advice were excluded from the study. All patients with ADHF having low Cr levels (<1.2 mg/dl) and no history of kidney disease who were managed in the coronary care unit during the same period were included as a control group.

### Plasma BNP levels

Our hospital authority has permitted the checking of serum BNP levels in all patients with heart failure since January 2006. Serum BNP levels were measured using the microparticle enzyme immunoassay test (Abbott, Chicago, IL, USA). In this study, all BNP levels were obtained within one hour after presentation at the emergency department or ICU.

### Clinical parameters

The medical records of the patients were reviewed, and the following information was recorded: age, gender, aetiology of CKD, prior history of congestive heart failure (CHF) and coronary artery disease, systolic/diastolic blood pressure, heart rate, admission diagnosis, blood urea nitrogen (BUN), serum Cr, estimated glomerular filtration rate (eGFR) from the abbreviated Modification of Diet in Renal Disease equation, C-reactive protein (CRP), echocardiographic data, sequential organ failure assessment (SOFA) and simplified acute physiology score II (SAPS II).

ADHF was defined as acute dyspnoea (New York Heart Association grade IV) plus overt pulmonary oedema on chest radiographs. To exclude lung diseases such as pneumonia and acute lung injury (ALI)/acute respiratory distress syndrome (ARDS), clinical course (or response to treatment) and final diagnosis at discharge were taken into account. A radiologist reviewed serial chest radiographs of all patients, and two other experts blinded to BNP results independently reviewed all available information and confirmed the final diagnoses. In cases of disagreement, two experts reviewed the cases together and reached a consensus.

ICU stay, ICU mortality, in-hospital mortality, all-cause death, new cardiac events and composite event rate (all-cause death and/or new cardiac event) were analysed. A new cardiac event was defined as the presence of any one of the following: readmission due to heart failure, acute coronary syndrome (ACS) or sudden death.

### Data acquisition and analyses

The association of BNP levels at admission with clinical and laboratory parameters, including biochemistry, left ventricular (LV) dysfunction and severity scores (SOFA and SAPS II), were investigated. Echocardiographic data were restricted to those obtained on the day of ICU admission when the correlations with BNP levels were analysed, but in the multivariate analysis for patient outcomes, all echocardiographic data obtained during the ICU stay were used.

The admission BNP levels were compared among three groups (CKD + ADHF, CKD - ADHF and controls) and the optimal cutoff level for differentiating CKD + ADHF from CKD - ADHF patients was chosen. ICU mortality and in-hospital mortality rates were calculated, and the effects of BNP levels at admission on these outcomes were investigated. Other significant factors affecting in-hospital mortality rate were also examined. For evaluation of the prognostic utility of admission BNP on long-term outcomes, the rates of all-cause death, new cardiac events and composite events were investigated. As an independent variable of admission BNP, continuous variables of BNP and BNP quartiles as well as the optimal cutoff level were used. As subgroup analysis, the data restricted to the dialysis-dependent patients were extracted and analysed separately.

### Statistical analyses

Data are expressed as the means ± standard deviation for continuous variables and as percentages for categorical variables unless otherwise indicated. For comparison of data between two groups, Student's *t*-test was used for continuous data and the chi-squared test was used for categorical data. Comparisons of data among three groups were performed using analysis of variance with Tukey's *post hoc *test. Correlation analyses were also performed using Pearson's correlations. However, for nonparametric data, the Mann-Whitney U test and Spearman correlation were applied. The ability of admission BNP to predict ADHF was assessed using receiver operating characteristic (ROC) curve analysis. For predicting in-hospital mortality, a multivariate logistic regression analysis was performed and for evaluating the effects of BNP level at admission on long-term outcome, Kaplan-Meier survival curves with log-rank tests and Cox regression analyses based on a multivariate approach were used. Variables with *P *< 0.10 on univariate analysis were included in multivariate analysis. *P *< 0.05 was considered statistically significant. All analyses were conducted using SAS statistical software, EG version (SAS Institute, Inc., Cary, NC, USA).

## Results

### Clinical data and admission BNP levels

The 236 patients with high Cr levels included 194 patients with CKD, and BNP levels at admission were available for 136 of them. The patients' baseline characteristics are shown in Table 1. Mean age was 66.5 ± 14.3 years and 66 (48.5%) patients were male. The aetiology of CKD was diabetes in 80 patients (58.8%), and 58 patients (42.6%) were dialysis dependent. The most common diagnosis on admission was ADHF (59.6%), followed by sepsis/infection (8.8%) and gastrointestinal/liver disease (7.4%).

**Table 1:** Baseline characteristics in CKD (n = 136) and control (n = 33) groups.

Variables	CKD group	Control group
Age (years)	66.5 ± 14.3	74.8 ± 10.4
Males/females	66/70	13/20
Aetiology of CKD		
Diabetes	80 (58.8%)	-
Hypertension	28 (20.6%)	-
Chronic glomerulonephritis	7 (5.1%)	-
Others/unknown	21 (15.4%)	-
BUN (mg/dL)	64.8 ± 34.9	22.2 ± 4.8
Serum Cr (mg/dL)	6.0 ± 4.1	0.9 ± 0.2
eGFR (ml/min/1.73 m^2^)	12.8 ± 7.3	71.4 ± 15.4
BNP (pg/mL)	1842.8 ± 1459.5	1418.9 ± 1126.5
Haemodialysis/peritoneal dialysis	47 (34.6%)/11 (8.1%)	
Prior history		
Congestive heart failure	35 (25.7%)	7 (21.2%)
Coronary artery disease	39 (28.7%)	9 (27.2%)
SAPS II	41.9 ± 13.4	29.7 ± 4.0
SOFA	5.8 ± 2.9	3.3 ± 3.2
Admission diagnosis		
ADHF	81 (59.6%)	33 (100%)
Sepsis/infection	12 (8.8%)	-
Gastrointestinal/liver	10 (7.4%)	-
Neurological disease	7 (5.1%)	-
Others	26 (19.1%)	-

ADHF = acute decompensated heart failure; BNP = brain natriuretic peptide; BUN = blood urea nitrogen; CKD = chronic kidney disease; Cr = creatinine; eGFR = estimated glomerular filtration rate; SAPS II = simplified acute physiology score II; SOFA = sequential organ failure assessment.

The association of BNP levels at admission with clinical and laboratory parameters are shown in Table 2. Systolic and diastolic blood pressure were correlated significantly with BNP levels at admission (*P *= 0.001 and *P *= 0.005, respectively). Echocardiography was performed in 87 patients (64.0%) with a median interval of one day (range, 0 to 7 days) after ICU admission. Among these patients, echocardiography was performed on the day of blood sampling for measuring admission BNP only in 45 patients (33.1%). Admission BNP levels showed significant correlations with LV ejection fraction (EF), LV diameter at end systole and LV mass index (LVMI; *P *= 0.008, *P *= 0.033 and *P *= 0.000, respectively), and the mean BNP level at admission in patients with LV systolic dysfunction (defined by EF <50%) was higher than in those without LV systolic dysfunction (2766.4 ± 1393.4 pg/ml vs. 1862.6 ± 1557.4 pg/ml, *P *= 0.032). In addition, patients who underwent mechanical ventilator (MV) treatment had higher BNP levels than those without MV treatment (2285.7 ± 1551.1 pg/ml vs. 1695.3 ± 1404.6 pg/ml, *P *= 0.041).

**Table 2:** Associations of BNP levels at admission with clinical and laboratory parameters.

Variables	Correlation coefficients	*P *value
Age	-0.146	0.090
BUN	-0.063	0.464
Serum Cr	0.013	0.884
Blood pressure		
Systolic blood pressure	0.297	0.001
Diastolic blood pressure	0.238	0.005
Heart rate	0.019	0.827
C-reactive protein	-0.074	0.407
WBC	-0.056	0.529
Haemoglobin	-0.015	0.859
Protein	0.104	0.230
Albumin	0.023	0.793
SAPS II	0.025	0.777
SOFA	0.099	0.257
Cardiac markers		
CK	0.067	0.454
CK-MB	0.056	0.529
Troponin I	0.066	0.460
Echocardiographic data		
LAD	0.277	0.076*
LV diameter at end systole	0.347	0.033*
LV diameter at end diastole	0.283	0.066*
LV mass index	0.526	0.000*
E/A ratio	-0.125	0.495*
Deceleration time	0.198	0.303*
LVEF	-0.391	0.008*
Systolic dysfunction (EF <50% *vs. *≥ 50%)	-	0.032^†^
Diastolic dysfunction (grade ≥ II *vs. *<I)	-	0.117^†^
Mechanical ventilation (yes *vs. *no)	-	0.041^#^

*Spearman correlation, ^†^Mann-Whitney U test, ^#^student's t test.

BNP = brain natriuretic peptide; BUN = blood urea nitrogen; CK = creatine kinase; CK-MB = creatinine kinase-MB; Cr = creatinine; E/A = ratio of E (peak mitral flow velocity of the early rapid filling) to A (peak velocity of the late filling due to atrial contration); EF = ejection fraction; LAD = left atrial dimension; LV = left ventricle; LVEF = left ventricular ejection fraction; SAPS II = simplified acute physiology score II; SOFA = sequential organ failure assessment; WBC = white blood cell.

### Diagnostic utility of BNP level at admission

**Figure 1:**
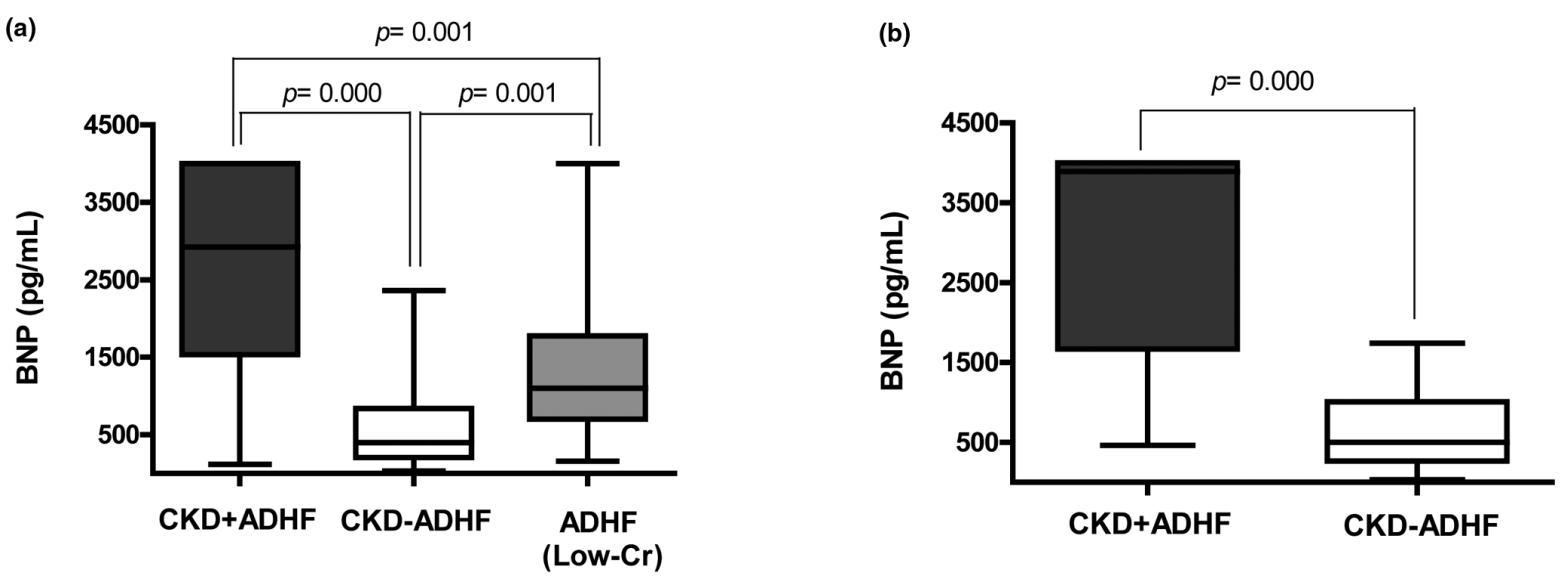
Comparison of BNP levels at admission.  **(a) **Brain natriuretic peptide (BNP) levels at admission among three groups: chronic kidney disease (CKD) + acute decompensated heart failure (ADHF) (mean ± standard deviation, 2708.6 ± 1246.9 pg/ml), CKD - ADHF (567.9 ± 491.7 pg/ml) and low-creatinine (Cr; <1.2 mg/dl)/ADHF (1418.9 ± 1126.5 pg/ml). BNP levels at admission were different among the three groups (*P *= 0.000, analysis of variance) and between each pair of groups (*P *= 0.001, *P *= 0.000 and *P *= 0.001, respectively, Tukey's *post hoc *test) with the highest mean value in the CKD + ADHF group. **(b) **BNP levels at admission of 58 dialysis-dependent patients with and without ADHF (3047.2 ± 1229.3 pg/ml vs. 632.3 ± 492.2 pg/ml, respectively, *P *= 0.000).

Eighty-one patients with CKD (59.6%) were diagnosed as having ADHF. The causes of ADHF were as follows: underdialysis (n = 38), ischemia (n = 20), infection (n = 13), arrhythmia (n = 5), uncontrolled hypertension (n = 4) and aortic dissection (n = 1). The results of eGFR were not significantly different between 81 patients with CKD + ADHF and 55 with CKD - ADHF (12.2 ± 6.5 ml/min/1.73 m^2 ^vs. 13.9 ± 8.2 ml/min/1.73 m^2^, respectively, *P *= 0.200). Mean BNP level at admission of 81 patients in the CKD + ADHF group was higher than that in the CKD - ADHF group (2708.6 ± 1246.9 pg/ml vs. 567.9 ± 491.7 pg/ml, *P *= 0.000) and was also higher than that in the control group (1418.9 ± 1126.5 pg/ml, *P *= 0.000; Figure 1a).

**Figure 2:**
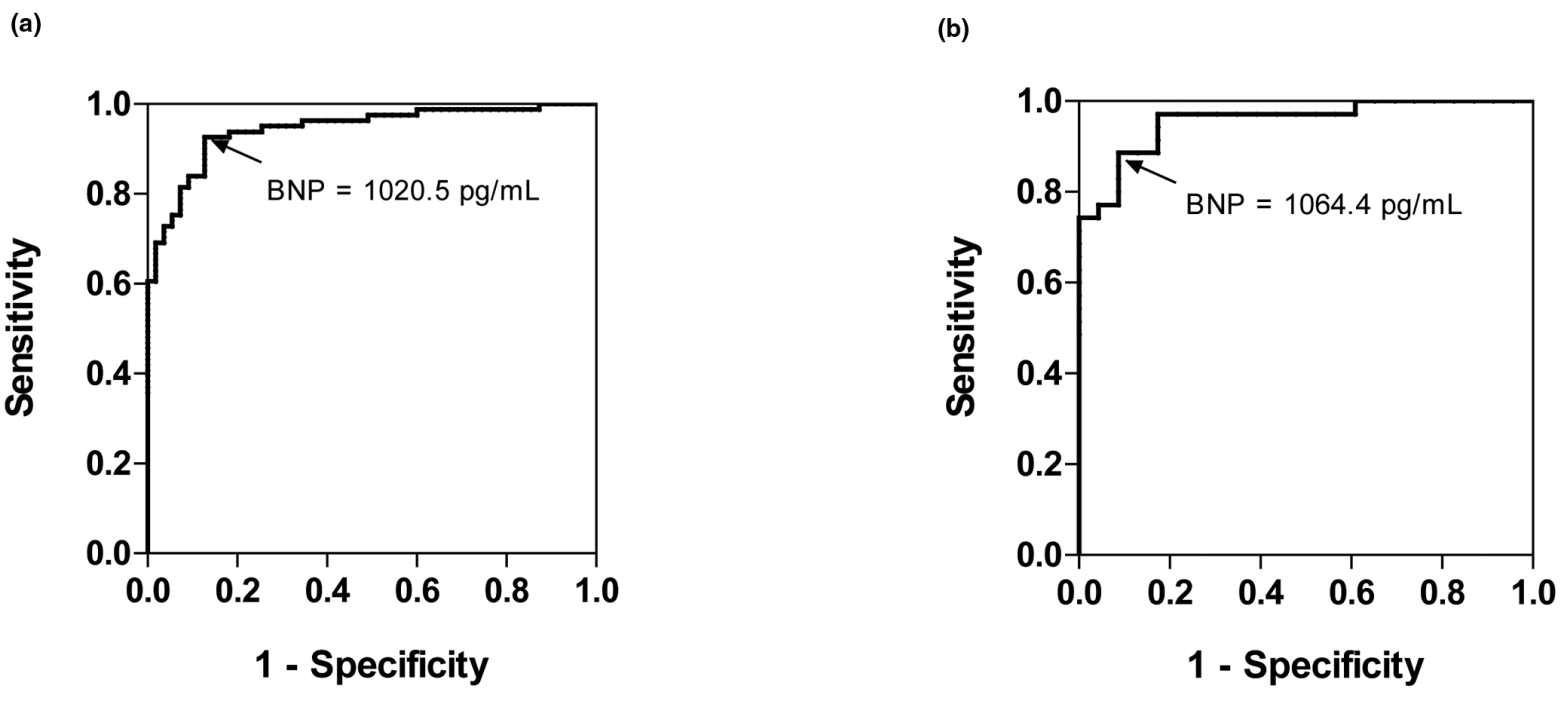
Receiver operating characteristic curves for BNP levels at admission for detecting ADHF.  **(a) **Receiver operating characteristic (ROC) curve for 136 patients with chronic kidney disease (CKD). The area under the curve (AUC) is 0.944 (95% confidence interval (CI), 0.907 to 0.981). The optimal cutoff level was estimated to be 1020.5 pg/ml. **(b) **ROC curve for 58 dialysis-dependent patients. The AUC value was 0.957 (95% CI, 0.909 to 0.999) and the optimal cutoff level was estimated to be 1064.4 pg/ml. BNP = brain natriuretic peptide.

The area under the ROC curve for BNP levels at admission to detect ADHF in patients with CKD was 0.944 (95% confidence interval (CI), 0.907 to 0.981) and the optimal cutoff value was 1020.5 pg/ml (Figure 2a). The BNP levels at admission representing a negative predictive value of 100% and a positive predictive value of 100% were 115.0 and 2382.5 pg/ml, respectively.

### BNP levels at admission and in-hospital outcomes

For the 136 patients with CKD for whom data were available, the median length of ICU stay was three days (range, 1 to 61 days), and ICU mortality and in-hospital mortality rates were 11.8% (16/136) and 16.2% (22/136), respectively. Univariate analysis indicated that BNP level at admission, BNP quartile ranges (482.8, 482.8 to 1401.0, 1401.0 to 3412.7 and >3412.7 pg/ml) and the optimal cutoff level (≥ 1020.5 pg/ml) had no effect on ICU stay, ICU mortality rate or in-hospital mortality rate. However, age, sex, BUN, systolic/diastolic blood pressure, albumin, CRP, creatine kinase, LV systolic dysfunction (EF <50%), SAPS II, SOFA and MV treatment were associated with in-hospital mortality (*P *< 0.10). Only MV treatment was an independent predictor of in-hospital mortality on multivariate logistic regression (odds ratio (OR) = 144.896, *P *= 0.042; Table 3).

**Table 3:** Univariate and multivariate analyses for predictors of in-hospital mortality

	Univariate anaysis	Multivariate analysis		
	
Variables	*P *value	*P *value	Odds ratios	95% confidence intervals
Age	0.007	0.137	1.145	0.958 to 1.368
Sex	0.049	0.402	0.387	0.042 to 3.573
BUN	0.025	0.545	1.011	0.977 to 1.045
Systolic blood pressure	0.022	0.603	0.990	0.951 to 1.029
Diastolic blood pressure	0.019	0.227	0.966	0.912 to 1.022
Albumin	0.007	0.967	0.960	0.067 to 13.701
C-reactive protein	0.000	0.797	0.998	0.983 to 1.013
CK	0.078	0.061	1.003	1.000 to 1.006
Systolic dysfunction (EF <50%)	0.085	0.208	5.125	0.402 to 65.388
SAPS II	0.000	0.556	0.936	0.749 to 1.168
SOFA	0.000	0.388	0.755	0.399 to 1.429
MV treatment	0.000	0.042	144.896	1.195 to 17573.2

BUN = blood urea nitrogen; CK = creatine kinase; EF = ejection fraction; SAPS II = simplified acute physiology score II; SOFA = sequential organ failure assessment; MV = mechanical ventilation.

### BNP level at admission and all-cause death, cardiac event and composite event rates

**Figure 3:**
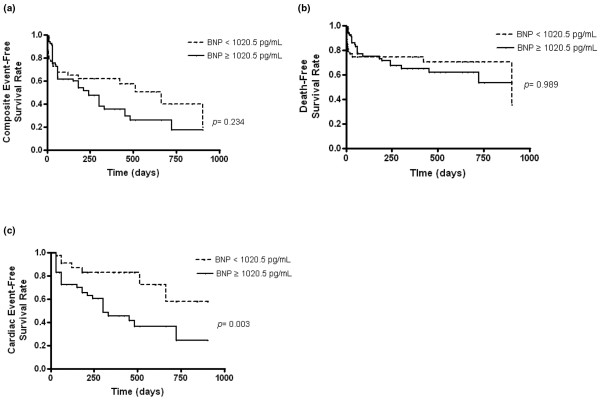
Kaplan-Meier curves for prognosis according to the optimal cutoff level of BNP levels at admission.  **(a, b) **No significant differences in composite event rate or all-cause death were observed between the two groups. **(c) **Patients with high brain natriuretic peptide (BNP) levels (≥ 1020.5 pg/ml) had significantly higher cardiac event rates during the follow-up period than those with low BNP levels (*P *= 0.003).

During the follow-up period for the 136 patients in the CKD group (median, 90 days; range, 1 to 900 days), 40 deaths and 38 new cardiac events (acute heart failure, n = 29; ACS, n = 7; sudden death, n = 2) occurred. High BNP levels above the optimal cutoff level (≥ 1020.5 pg/ml) were not associated with composite event-free survival or death-free survival on Kaplan-Meier curves (log rank, *P *= 0.234 and *P *= 0.989, respectively; Figures 3a and 3b), but was significantly related to a higher rate of new cardiac events (log rank, *P *= 0.003) during the follow-up period (521.1 ± 44.7 days; Figure 3c).

Univariate Cox analysis indicated that white blood cells, serum Cr, LVEF, left atrial dimension (LAD), LVMI, CRP, ADHF and high BNP levels (≥ 1020.5 pg/ml) were associated with the occurrence of new cardiac events (*P *< 0.10; Table 4). In the multivariate Cox model, serum Cr and LAD were significantly associated with new cardiac events (hazard ratio (HR) = 0.855, *P *= 0.046; HR = 1.083, *P *= 0.034, respectively) and a high BNP level above the optimal cutoff level (≥ 1020.5 pg/ml) showed a borderline significant association (HR = 4.551, *P *= 0.078). However, a significant association was observed when continuous variables of BNP level or BNP quartile, instead of optimal cutoff level, were used in the multivariate Cox model (HR = 1.001, *P *= 0.041; HR = 2.212, *P *= 0.018, respectively).

**Table 4:** Univariate and multivariate analyses for predictors of new cardiac events

	Univariate analysis	Multivariate analysis		
	
Variables	*P *value	*P *value	Hazard ratios	95% confidence intervals
WBC	0.053	0.410	1.000	1.000 to 1.000
Serum Cr	0.044	0.046	0.855	0.734 to 0.997
LVEF (%)	0.010	0.842	0.997	0.964 to 1.031
LAD	0.004	0.034	1.083	1.006 to 1.167
LV mass index	0.087	0.606	1.002	0.994 to 1.011
C-reactive protein	0.066	0.890	0.999	0.988 to 1.010
ADHF	0.038	0.780	0.816	0.197 to 3.385
Admission BNP				
Optimal cutoff level*	0.007	0.078	4.551	0.845 to 24.502
BNP (continuous variable)	0.005	0.041	1.001	1.0002 to 1.0010
BNP quartiles	0.005	0.018	2.212	1.145 to 4.276

*BNP ≥ 1020.5 pg/mL.

ADHF = acute decompensated heart failure; BNP = blood natriuretic peptide; Cr = creatinine; LAD = left atrial dimension; LV = left ventricular; LVEF = left ventricular ejection fraction; WBC = white blood cells.

### Dialysis-dependent patients

Of the 136 patients with CKD, 58 (42.6%) were dialysis dependent. In this subgroup, admission BNP levels in the CKD + ADHF group (n = 35, 60.3%) were higher than those in the CHF - ADHF group (3047.2 ± 1229.3 pg/ml vs. 632.3 ± 492.2 pg/ml, *P *= 0.000; Figure 1b). The area under the ROC curve was 0.957 (95% CI, 0.909 to 0.999) and the optimal cutoff level was estimated to be 1064.4 pg/ml (Figure 2b). High BNP levels (≥ 1064.4 pg/ml) had no effect on in-hospital mortality, all-cause mortality or composite-event rate during the follow-up period (507 ± 71 days). The patients with high BNP levels on admission (≥ 1064.4 pg/ml) had a significantly higher new cardiac event rate than those with low BNP levels on Kaplan-Meier curves (log rank, *P *= 0.026), and the HR was 4.39 (*P *= 0.05) on univariate Cox analysis.

## Discussion

The results of the present study showed that BNP levels at admission in patients with CKD + ADHF were different from those with CKD - ADHF and low-Cr/ADHF. High BNP was associated with LV systolic dysfunction and was helpful in differentiating patients who had ADHF from those without ADHF among those with CKD in the ICU setting. Although admission BNP was not associated with in-hospital mortality rate, it was an independent predictor of new cardiac events during the follow-up period in patients with CKD admitted to the ICU. According to the recent classification of CRS [4,5], the present study indicates that BNP levels have the diagnostic and prognostic capability for CRS type 4 in ICU patients.

Although the value of BNP has been previously investigated in patients with CKD, most studies were conducted using stable outpatients and little information is available regarding patients with CKD in the ICU setting [13,15-19]. Considering the frequent elevation of BNP levels in the ICU setting due to critical illnesses, for example, sepsis [20,21], acute exacerbation of chronic respiratory failure [22] and ALI/ARDS [23,24], it seems to be more difficult to interpret the measured BNP levels in patients with CKD. However, because this patient group is still at high risk of volume overload (pulmonary oedema) and cardiovascular mortality [25,26], investigation of the BNP value may also be important in patients with CKD in the ICU setting.

Among the clinical and laboratory parameters examined in this study, high systolic/diastolic blood pressure and LV systolic dysfunction (EF <50%) were significantly associated with high BNP levels at admission. However, in contrast to previous studies, age, diabetes and eGFR were not associated with BNP levels at admission [15,16,18]. These discrepancies may have been due not only to the high proportion of patients with ADHF (59.6%) and dialysis-dependent patients (42.6%) but also to various critical illnesses causing increases in BNP levels in our study population.

Recently, a few studies of BNP levels among ICU patients with pulmonary oedema have been reported. In a study of 81 ICU patients, Karmpaliotis and colleagues reported a median BNP level in patients with cardiogenic pulmonary oedema of 1260 pg/ml (interquartile range (IQR), 540 to 2020 pg/ml) vs. 325 pg/ml (IQR, 82 to 767 pg/ml) in those with ALI/ARDS, and Levitt and colleagues reported mean values in these groups of 747 ± 476 pg/ml and 496 ± 439 pg/ml, respectively, in a study of 54 critically ill patients [23,24]. The BNP levels in our series were higher than in these previous studies, both in patients with pulmonary oedema (ADHF) and without. However, in the control group (with ADHF and low Cr), the mean BNP level was similar to that in patients with pulmonary oedema reported by Karmpaliotis and colleagues. Considering the similar eGFR levels between CKD + ADHF and CKD - ADHF groups, we cannot suggest that the higher BNP levels in the CKD + ADHF group may have been caused by renal dysfunction rather than by pulmonary oedema (i.e., fluid overload causing cardiac stress). However, these observations indicated that both cardiac and renal dysfunction were crucial factors affecting BNP levels, and a significant difference in BNP level was observed between patients with CKD + ADHF and CKD - ADHF despite the frequent high levels of BNP in patients with CKD in the ICU setting.

The optimal cutoff BNP level for differentiating between patients with CKD + ADHF and those with CKD - ADHF was estimated to be 1020.5 pg/ml. However, several arguments can be made against the diagnostic utility of BNP levels in patients with CKD in the ICU due to the many confounding factors beyond renal and cardiac dysfunction in the ICU setting and because the diagnosis of ADHF can be made without BNP levels. Based on the findings of this study, patients with high BNP levels are likely to have LV systolic dysfunction, and the BNP level at admission may be a useful tool for detecting ADHF in patients with CKD in the ICU setting. This possibility should be investigated in a future large-scale prospective study.

In terms of hospital outcome, admission BNP was not associated with ICU stay, ICU mortality rate or in-hospital mortality rate in this study, and MV treatment was the only significant factor predicting in-hospital mortality in 136 patients with CKD for whom BNP level data were available. Unfortunately, very little information is available on hospital outcomes in patients with CKD admitted to the ICU to which we can either reference or compare our results (in-hospital mortality rate, 16.2%). Based on this study, hospital outcomes were not dependent on BNP level at admission, but rather some other factor(s).

With regard to the prognostic role of BNP level, several studies support the utility of BNP in the long-term prognosis of patients with CKD; these studies consistently demonstrated a positive relation of BNP levels to long-term outcomes, such as all-cause death and cardiac events [13,16,18,27,28]. In these studies, however, the subjects were mostly stable outpatients at study entry, while our study focused on those admitted to the ICU due to acute illness. The significant relation revealed by multivariate analyses in our study between higher BNP levels and the incidence of new cardiac events indicate that BNP level, even when measured in the unstable state as in the ICU setting, could be a useful marker for predicting future cardiac events.

BNP levels are usually elevated in both haemodialysis and peritoneal dialysis patients [29-32]. In subgroup analysis with dialysis-dependent patients, this study demonstrated a similar cutoff level to that of all 136 patients with CKD, and high admission BNP levels were also associated with a high incidence of new cardiac events, which was consistent with previous studies [16,27,28]. Although no large-scale trials have been performed on dialysis-dependent patients, these results suggest that BNP levels at admission may still be a useful tool regardless of dialysis state in the ICU.

This study had several limitations. First, this was a retrospective study and the number of patients was limited. Therefore, the data may reflect some unintended bias. In particular, of 194 patients with CKD, 58 patients were excluded due to the absence of BNP results. This exclusion might have led to an increased number of patients with ADHF and the elevated BNP levels. Second, the diagnosis of ADHF in this study was made mainly by clinical and radiological findings. However, in clinical practice, patients with ADHF may not have pulmonary oedema in chest radiographs and in some patients with ADHF the radiographic findings are similar to those of ALI/ARDS. Therefore, some misclassification may have occurred in this study. Third, the data for subsequent deaths and cardiac events were based on electronic records. Therefore, even with high adherence of patients with CKD to the institution, outcomes may have been missed. Fourth, we could not investigate the changes in BNP level during ICU stay or BNP levels at discharge, which may reflect the effectiveness of ICU treatment and be associated with patient outcomes [33]. This was mainly due to the nature of this study, which did not have a prognostic design. Fifth, we could not evaluate the degree of patient compliance with prescribed medications, which could have a major impact on clinical outcomes. Despite these limitations, to our knowledge, this is the first study on the clinical utility of BNP levels in patients with CKD in the ICU setting.

## Conclusions

In this study, BNP levels at admission in patients with CKD in the ICU were not associated with in-hospital mortality. However, BNP levels at admission was useful for detecting ADHF and predicting future cardiac events (i.e., CRS type 4) in patients with CKD admitted to the ICU. Therefore, despite various confounding factors, BNP levels at admission may be useful even in critically ill patients with CKD admitted to the ICU. Further prospective studies with larger cohorts are needed to define the precise role of BNP in patients with CKD in the ICU setting.

## Key messages

• BNP levels at admission in the CKD + ADHF group were significantly higher than those in the CKD - ADHF and low-Cr/ADHF groups.

• High BNP level was associated with LV systolic dysfunction and may be helpful for detecting ADHF in patients with CKD in the ICU setting.

• High BNP levels at admission was not associated with in-hospital mortality, but were significantly associated with new cardiac events in patients with CKD admitted to the ICU.

• Despite various confounding factors, BNP levels at admission may be a useful marker for CRS type 4 in ICU patients.

## Abbreviations

ACS: acute coronary syndrome; ADHF: acute decompensated heart failure; ALI: acute lung injury; ARDS: acute respiratory distress syndrome; BNP: brain natriuretic peptide; BUN: blood urea nitrogen; CHF: congestive heart failure; CI: confidence interval; CKD: chronic kidney disease; Cr: creatinine; CRP: C-reactive protein; CRS: cardio-renal syndrome; EF: ejection fraction; eGFR: estimated glomerulofiltration rate; ICU: intensive care unit; IQR: interquartile range; LAD: left atrial dimension; LV: left ventricle; LVMI: left ventricular mass index; MV: mechanical ventilation; OR: odds ratio; ROC: receiver operating characteristic; SAPS II: simplified acute physiology score II; SOFA: sequential organ failure assessment.

## Competing interests

The authors declare that they have no competing interests.

## Authors' contributions

SP conducted the study, performed data collection and statistical analysis, and drafted the manuscript. GYC reviewed and collected echocardiographic data, and revised the manuscript critically. SGK participated in the design of the study and revised the manuscript critically. YIH, HRK, SHJ, DGK and YRS participated in the design and coordination of the study. YAB reviewed chest radiography. KSJ conceived of the study and participated in its design and coordination, and revised the manuscript critically. All authors read and approved the final manuscript.
